# Supplementary material for: Effect of apolipoprotein genotype and educational attainment on cognitive function in autosomal dominant Alzheimer’s disease
Source: Nat Commun. 2023 Aug 23;14:5120. doi: 10.1038/s41467-023-40775-z (PMC10447560; doi:10.1038/s41467-023-40775-z)
Supplement: Supplementary file 1 — Supplementary Information [file 41467_2023_40775_MOESM1_ESM.pdf]

Supplementary Materials for

Effect of apolipoprotein genotype and educational attainment on cognitive function in autosomal dominant Alzheimer's disease due to the Presenilin-1 E280A mutation

Supplementary Tables

Supplementary Table 1

Age of clinical onset of MCI or dementia in *PSEN1* E280A carriers by stratified by APOE genotype

	**APOE e4+**	**APOE e4-**	***p*-value**
**MCI onset**	43.20±5.56 (n=64)	45.09±6.37 (n=201)	.009
**Dementia onset**	47.45±5.15 (n=60)	49.17±5.13 (n=173)	.019
	**APOE e2+**	**APOE e2-**	***p*-value**
**MCI onset**	45.65±7.90 (n=31)	44.50±5.98 (n=234)	.084
**Dementia onset**	49.64±4.49 (n=25)	48.62±5.28 (n=208)	.092

Means and standard deviations given; number of participants per group in parentheses; uncorrected *p* values from two-sided Mann-Whitney U tests

Supplementary Table 2

Participant demographics stratified by *PSEN1* and APOE genotype

	**PSEN1 E280A Carriers**			**PSEN1 E280A Non-Carriers**		
	**APOE e2 +**	**APOE e2 -**	***p***	**APOE e2 +**	**APOE e2 -**	***p***
**N**	102	573		73	521	
**Age**	35.40±11.55	33.91±11.12	0.20	33.93±11.45	25.15±12.07	0.47
**Sex (M/F)**	44/58	261/312	0.65	26/47	236/285	0.12
**Educational attainment (years)**	7.33±4.42	7.45±4.38	0.69	7.97±4.81	8.27±4.69	0.70
**Mini Mental State Examination**	27.28±4.25	26.33±5.53	0.37	28.67±1.99	28.76±2.12	0.66

Means and standard deviations given for age, educational attainment, and Mini Mental State Examination; uncorrected *p* values from two-sided statistical comparisons (Mann-Whitney U tests used to compare age, educational attainment, and Mini Mental State Examination scores; chi-square test used to compare sex)

Supplementary Figures

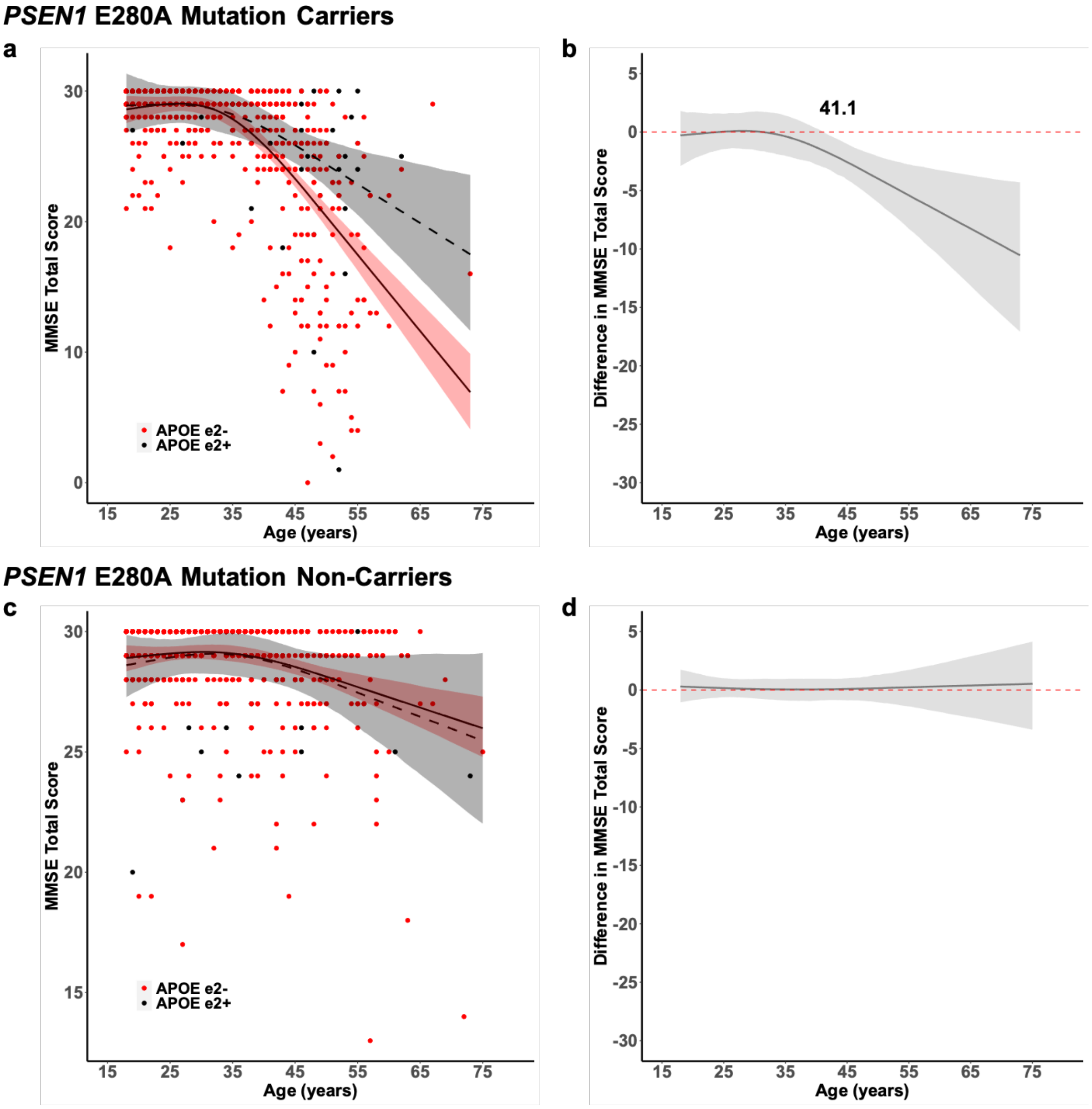

Supplementary Figure 1. **Age-related trajectories of cognitive impairment in *PSEN1* E280A mutation carriers and non-carriers stratified by presence or absence of APOE e2**. **A** Cross-sectional MMSE scores of *PSEN1* E280A mutation carriers who are APOE e2+ (black) and APOE e2- (red) as a function of age. **b** Differences in MMSE score between APOE e2+ and e2- *PSEN1* E280A mutation carriers as a function of age. MMSE score declines in APOE e2+ *PSEN1* E280A mutation carriers begins to differ from APOE e2- *PSEN1* E280A mutation carriers at 41.1 years. **c** Cross-sectional MMSE scores of *PSEN1* E280A mutation non-carriers who are APOE e2+ (black) and APOE e2- (red) as a function of age. **d** Differences in MMSE score between APOE e2+ and e2- *PSEN1* E280A mutation non-carriers as a function of age. MMSE score does not differ between APOE e2+ and e2- *PSEN1* E280A mutation non-carriers in this age range. The shaded areas of each plot represent the 99% credible intervals around the model estimates drawn from the distributions of model fits derived by the Hamiltonian Markov chain Monte Carlo analyses. MMSE = Mini Mental State Examination. Source data are provided as a Source Data file.

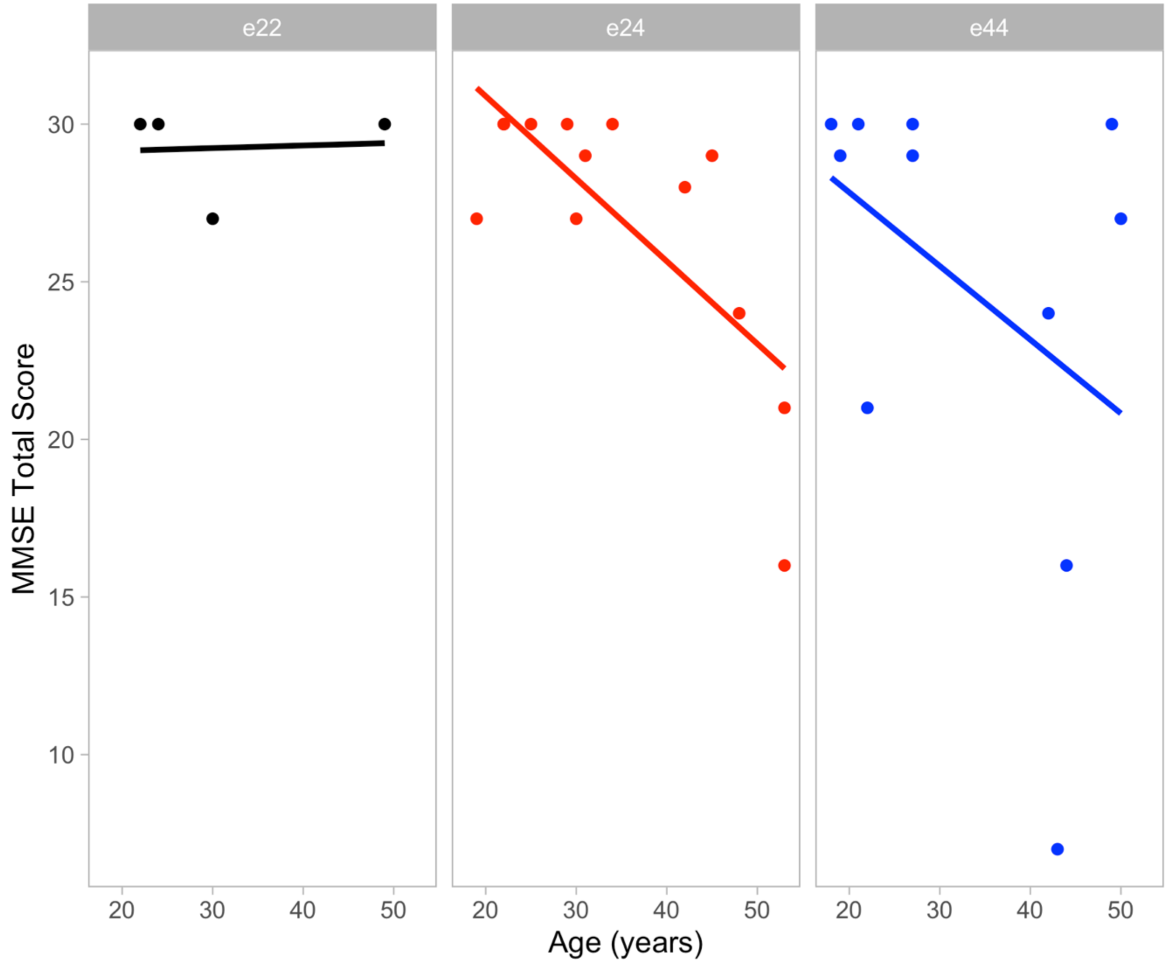

Supplementary Figure 2. Cross-sectional MMSE scores of *PSEN1* E280A mutation carriers who are APOE e2/2 (black), APOE e2/4 (red), and APOE e4/4 (blue) as a function of age.

Supplementary Analysis

Role of urban and rural living conditions

Because an association between ruralness and educational attainment has previously been reported in *PSEN1* E280A carriers, we performed a series of analyses to determine the role of urban and rural living conditions in our cohort. A subset of *PSEN1* E280A carriers had available data on urban versus rural living conditions:

	**APOE e4+ (n = 124)**	**APOE e4- (n = 477)**
**Rural (n = 171)**	44 (35%)	127 (27%)
**Urban (n = 430)**	80 (65%)	350 (73%)

*chi-square *p*-value = .051

PSEN1 carriers who lived in urban areas had on average more years of educational attainment than those who lived in rural areas (urban average: 7.70 years, rural average: 6.90 years; p = .033). Because type of living area was associated with years of educational attainment, we next assessed whether type of living had similar associations with cognition and APOE genotype as observed with educational attainment. We conducted a linear regression with type of living, APOE e4 genotype, and their interaction to predict MMSE scores in *PSEN1* carriers. APOE e4 genotype remained a statistically significant predictor of MMSE score (p = .043), but type of living (p = .080) and the interaction term between type of living and APOE genotype (p = .069) did not reach significance.

To assess potential confounds with age, we then examined its associations with educational attainment and type of living. Age was negatively associated with years of educational attainment (r = -0.33, p < .001). The proportion of rural versus urban living in “young” (18-30 years) and “old” (31+ years) *PSEN1* carriers was similar:

	**Young (n = 267)**	**Old (n = 334)**
**Rural (n = 171)**	73 (27%)	98 (29%)
**Urban (n = 430)**	194 (73%)	236 (71%)

* chi-square *p*-value = .589
