# Supplementary material for: Effect of apolipoprotein genotype and educational attainment on cognitive function in autosomal dominant Alzheimer’s disease
Source: Nat Commun. 2023 Aug 23;14:5120. doi: 10.1038/s41467-023-40775-z (PMC10447560; doi:10.1038/s41467-023-40775-z)
Supplement: Supplementary file 3 — Reporting Summary [file 41467_2023_40775_MOESM3_ESM.pdf]

nature portfolio

Corresponding author(s): Dr. Yakeel T. Quiroz

Last updated by author(s): Jul 14, 2023

Reporting Summary

Nature Portfolio wishes to improve the reproducibility of the work that we publish. This form provides structure for consistency and transparency in reporting. For further information on Nature Portfolio policies, see our Editorial Policies and the Editorial Policy Checklist.

Statistics

For all statistical analyses, confirm that the following items are present in the figure legend, table legend, main text, or Methods section.

n/a	Confirmed	
☐	☒	The exact sample size (*n*) for each experimental group/condition, given as a discrete number and unit of measurement
☐	☒	A statement on whether measurements were taken from distinct samples or whether the same sample was measured repeatedly
☐	☒	The statistical test(s) used AND whether they are one- or two-sided<br>*Only common tests should be described solely by name; describe more complex techniques in the Methods section.*
☐	☒	A description of all covariates tested
☐	☒	A description of any assumptions or corrections, such as tests of normality and adjustment for multiple comparisons
☐	☒	A full description of the statistical parameters including central tendency (e.g. means) or other basic estimates (e.g. regression coefficient) AND variation (e.g. standard deviation) or associated estimates of uncertainty (e.g. confidence intervals)
☐	☒	For null hypothesis testing, the test statistic (e.g. *F*, *t*, *r*) with confidence intervals, effect sizes, degrees of freedom and *P* value noted<br>*Give P values as exact values whenever suitable.*
☐	☒	For Bayesian analysis, information on the choice of priors and Markov chain Monte Carlo settings
☒	☐	For hierarchical and complex designs, identification of the appropriate level for tests and full reporting of outcomes
☒	☐	Estimates of effect sizes (e.g. Cohen's *d*, Pearson's *r*), indicating how they were calculated

Our web collection on statistics for biologists contains articles on many of the points above.

Software and code

Policy information about availability of computer code

Data collection	Cognitive data were stored using REDCap version 13.1.29. No software was used in data collection.
Data analysis	Data analysis was conducted in R version 4.2.0

For manuscripts utilizing custom algorithms or software that are central to the research but not yet described in published literature, software must be made available to editors and reviewers. We strongly encourage code deposition in a community repository (e.g. GitHub). See the Nature Portfolio guidelines for submitting code & software for further information.

Data

Policy information about availability of data

All manuscripts must include a data availability statement. This statement should provide the following information, where applicable:

- Accession codes, unique identifiers, or web links for publicly available datasets
- A description of any restrictions on data availability
- For clinical datasets or third party data, please ensure that the statement adheres to our policy

The data analyzed in this study are protected and are not publicly available in full to protect the identities of the members of this kindred. The datasets will be made available from the corresponding author on reasonable request. Source data are provided with this paper as a Source Data file.

Human research participants

Policy information about studies involving human research participants and Sex and Gender in Research.

Reporting on sex and gender	Sex was determined based on participant self-report. Male/female distribution is reported for each group of interest in the manuscript (Table 2). Overall, 567 males and 702 females were included in this study. Sex was not considered as a variable of interest in these analyses due to no a priori hypotheses about sex differences and sample size limitations. Groups of interest did not differ in proportion of males/females. All participants with available data for the study aims were included in analyses regardless of sex/gender.
Population characteristics	Participants are adult members of a kindred residing in Colombia with a high prevalence of the Presenilin-1 E280A mutation. Additional details are included in the "Behavioural & social sciences study design" section.
Recruitment	Participants were identified from the pre-existing Alzheimer's Prevention Initiative (API) registry of autosomal dominant Alzheimer's disease. The API includes more than 6,000 living members of a kindred with a high prevalence of the Presenilin-1 E280A mutation. All members of the registry reside in Colombia and have a parent with the Presenilin-1 E280A mutation, but all members are blind to their own genetic status. Because all participants have a parent with the Presenilin-1 E280A mutation, it is unlikely to be a source of bias in our results; however, it is possible that there are other self-selection biases in an individual's decision to participate in research. Both participants and investigators were blind to genetic status during data collection to avoid introducing potential bias into results. Potential sources of bias from inclusion criteria (adult age, data availability) is unlikely to impact our results.
Ethics oversight	Study procedures were approved by the Institutional Review Board of the University of Antioquia in Colombia (21-10-605) and were performed in accordance with the ethical standards of the Declaration of Helsinki. All participants provided informed consent prior to the initiation of study procedures. Participants were compensated for their participation in accordance with the approved guidelines.

Note that full information on the approval of the study protocol must also be provided in the manuscript.

Field-specific reporting

Please select the one below that is the best fit for your research. If you are not sure, read the appropriate sections before making your selection.

☐ Life sciences ☒ Behavioural & social sciences ☐ Ecological, evolutionary & environmental sciences

For a reference copy of the document with all sections, see nature.com/documents/nr-reporting-summary-flat.pdf

Behavioural & social sciences study design

All studies must disclose on these points even when the disclosure is negative.

Study description	This is an observational study of quantitative, cross-sectional, retrospective data
Research sample	Participants are adult members (18 to 75 years old) of a kindred residing in Colombia with a high prevalence of the Presenilin-1 E280A mutation. In total, 675 Presenilin-1 E280A mutation carriers and 594 mutation non-carriers were included in analyses. Overall, 567 males and 702 females were included in this study. Participants included in the study are representative of adults living in Antioquia, Colombia. Participants were identified through the Alzheimer's Prevention Initiative (API) registry of autosomal dominant Alzheimer's disease. This sample was chosen because of the high prevalence of the PSEN1 E280A mutation and for the high degree of environmental matching of the mutation non-carriers.
Sampling strategy	Participants were initially recruited into the Alzheimer's Prevention Registry for autosomal dominant Alzheimer's disease. Participants for this study were identified from a study previously published by YTQ and further filtered to include only participants who were over the age of 18 and had available APOE genotype and cognitive data. Because this study uses retrospective data, we did not perform an a priori sample size calculation. We included all participants that met the inclusion criteria. This resulted in a larger sample size than prior studies who have investigated effects of APOE in the PSEN1 kindred, which is sufficient for our study aims.
Data collection	Participants and study personnel were blind to genetic status during data collection. Genetic information was obtained via blood draw. Neuropsychological assessments were completed using pen and paper and stored on a computer database (REDCap). Only the investigators and research participants were present during data collection.
Timing	Data collection occurred between 2001 and 2017.
Data exclusions	No data were excluded after arriving at the sample as described above.
Non-participation	N/A: Because this is a retrospective study, all participants who met our inclusion criteria were included in analyses.
Randomization	Participants were grouped based on their genotype (PSEN1, APOE). Research participants and study personnel were blind to genetic

status until data analysis.

Reporting for specific materials, systems and methods

We require information from authors about some types of materials, experimental systems and methods used in many studies. Here, indicate whether each material, system or method listed is relevant to your study. If you are not sure if a list item applies to your research, read the appropriate section before selecting a response.

Materials & experimental systems

n/a	Involved in the study
☒	☐ Antibodies
☒	☐ Eukaryotic cell lines
☒	☐ Palaeontology and archaeology
☒	☐ Animals and other organisms
☒	☐ Clinical data
☒	☐ Dual use research of concern

Methods

n/a	Involved in the study
☒	☐ ChIP-seq
☒	☐ Flow cytometry
☒	☐ MRI-based neuroimaging
